# Research progress on the role of mindfulness in intervention for adolescent obesity

**DOI:** 10.3389/fendo.2024.1412522

**Published:** 2024-08-21

**Authors:** Shuming Shao, Linda Li, Yimin Zhang, Zheng Liu, Xiaorui Zhang

**Affiliations:** ^1^ Department of Pediatrics, Peking University People’s Hospital, Beijing, China; ^2^ Department of Clinical Medicine, Peking University Health Science Center, Beijing, China; ^3^ Department of Maternal and Child Health, School of Public Health, Peking University, Beijing, China

**Keywords:** mindfulness, intervention, obesity, adolescent obesity, research progress

## Abstract

In the face of the increasingly serious background of overweight and obesity rates among adolescents in China, mindfulness, as an emerging therapeutic approach, has shown its unique effectiveness. This article reviewed the research progress of mindfulness in the intervention of adolescent obesity, summarized its effects on improving physiological and psychological indicators, and listed the different options for implementing mindfulness therapy. These studies supported the preliminary effectiveness of mindfulness in the intervention of adolescent obesity, providing a basis for mindfulness to become a new approach for obesity intervention in the future.

## 1 Introduction

In recent years, as living standards have improved and dietary habits have shifted, the prevalence of overweight and obesity among children and adolescents in China has been increasing rapidly. According to the “Report on the Nutrition and Chronic Diseases Status of Chinese Residents (2020)” (1), the rate of overweight and obesity among children and adolescents aged 6 to 17 has reached 19.0%. Multiple factors contribute to adolescent obesity, with unreasonable diet and sedentary behavior being the main causes. Childhood overweight and obesity are associated with long-term chronic diseases (2). The prevention and control of obesity in children and adolescents has gradually become a serious public health issue. In addition to conventional intervention methods such as adjusting dietary structure, engaging in physical exercise, and providing health education, recent studies have discovered that mindfulness, as an emerging intervention method, can be applied to the intervention of adolescent obesity and achieve positive results (3). This article will review the research progress of applying mindfulness in the intervention of adolescent obesity.

## 2 Concept and methods of mindfulness

Mindfulness, stemming from Buddhist meditation, is widely regarded in the present era as the intention and non-judgmental focus on present moment awareness, typically achieved through meditation and yoga. Intention involves consciously making a decision to do something, while non-judgmental focus involves maintaining attention on a specific object, present moment awareness involves thoughts, feelings, emotions, behaviors, or environments that exist in the present moment, emphasizing the current experience rather than dwelling on the past. The concept of Mindfulness-Based Intervention was first introduced to American medical institutions in 1979 by Jon Kabat-Zinn, who pioneered an 8-week program called Mindfulness-Based Stress Reduction, which included formal meditation practices such as body scans, sitting meditation, and hatha yoga, as well as informal practices that integrate mindfulness into daily tasks, including walking, standing, and eating (4). Mindfulness-Based Stress Reduction was initially designed to assist patients in alleviating stress and managing pain. Through research, it has been discovered that this approach has broad applications in the treatment of anxiety, depression, insomnia, hypertension, eating disorders, cancer-related pain, and other conditions (5–9). As a result, various mindfulness-based therapies have emerged in recent years, such as Mindfulness-Based Cognitive Therapy, Dialectical Behavior Therapy and Mindfulness-Based Eating Awareness Training (MB-EAT). These therapies have been widely utilized in the treatment of various mental and physical health conditions (8, 10).

Mindfulness-Based Cognitive Therapy is based on Mindfulness-Based Stress Reduction and incorporates the concepts of cognitive behavioral therapy. It aims to improve low mood and negative thoughts through meditation practices, and is often used for preventing depressive disorder (11). There is evidence suggesting that Mindfulness-Based Cognitive Therapy can effectively relieve the symptoms of depression and anxiety, and lower the relapse rate in those who are more vulnerable to depression (12). Moreover, Mindfulness-Based Cognitive Therapy can function as an effective intervention measure for patients with somatic dysmorphic disorder, improving their symptoms, emotional regulation disorders, and executive function (13).

On the other hand, Dialectical Behavior Therapy, is also a mindfulness-based therapy. Dialectical Behavior Therapy was originally developed as an intervention measure for patients who meet the criteria for borderline personality disorder and is described as involving acceptance and change. It encourages clients to honestly accept themselves, their history, and their current situation, while striving to change their behavior and environment to build a better life. Dialectical Behavior Therapy includes a wide range of cognitive and behavioral therapy programs, most of which are aimed at changing thoughts, emotions, or behaviors (11). Standard Dialectical Behavior Therapy consists of four core components: individual therapy, group skills training, telephone counseling, and therapist counseling teams (14). In a randomized clinical trial, Dialectical Behavior Therapy demonstrated efficacy in reducing suicide attempts in high-risk populations of adolescents with bipolar disorder (15).

In addition, in adolescent obesity intervention, mindful eating is also an important method. The concept of mindful eating involves focusing on the food being consumed at the moment, recognizing hunger and fullness cues, consciously choosing food, slowing down the eating pace, utilizing all sensory experiences during eating, and paying attention to bodily signals. This practice enhances awareness and self-regulation of hunger, fullness, taste satisfaction, eating speed, and emotions (16). These skills play a crucial role in changing the relationship between individuals and food, which can enhance autonomous motivation and self-regulation ability, reduce cravings and desires for food, and are therefore used to treat eating disorders and assist with weight management (17). A well-established mindfulness eating therapy is called MB-EAT, which incorporates mindfulness meditation, mindful eating, emotional awareness, and self-acceptance. The goal of MB-EAT is to cultivate awareness of internal and external cues related to eating, break the cycle of binge eating, self-blame, and dysfunctional restraint, and restore the natural physiological processes of eating regulation. Moreover, this therapy emphasizes the nutritional value and enjoyment that food provides, thereby encouraging healthier food choices in terms of types and quantities to achieve weight control goals (18). An adolescent-adapted MB-EAT program developed by Barnes et al. (19) has confirmed the feasibility of implementing this approach among teenagers.

## 3 The role of mindfulness in adolescent obesity intervention

In recent years, mindfulness intervention has been gradually applied to adolescent obesity intervention, and there have indeed been some relevant studies conducted. Existing research suggests that many indicators can reflect the effectiveness of adolescent obesity intervention, and body mass index (BMI) and waist-hip ratio (WHR) are the most directly relevant indicators among term (20, 21). Physiological indicators such as blood pressure, heart rate, and serum cortisol levels can reflect the changes in the physical function of obese patients (22). In addition, dietary choices, exercise habits, impulsivity, eating disorders, and the psychological well-being of adolescents are all related to the onset of adolescent obesity (19, 23, 24). An improvement in these indicators indicates the positive intervention effects of mindfulness.

### 3.1 The role of mindfulness in reducing BMI and WHR

BMI is calculated by dividing weight (in kilograms) by the square of height (in meters). The waist-to-hip ratio is determined by dividing the waist circumference by the hip circumference. BMI primarily assesses overall obesity based on body density, while WHR focuses central obesity (21). BMI can be used as a direct indicator to evaluate the effectiveness of obesity intervention. Daly et al. (20) conducted a randomized controlled trial with 37 adolescent females aged 14 to 17, with a BMI above the 90th percentile. They found that compared to the control group, which received conventional diet and exercise, the mindfulness intervention group experienced an average BMI reduction of 1.1 kg/m^2^ after a 6-week intervention (p < 0.001), while the control group increased by 0.7 kg/m^2^. Stavrou et al. (25) also demonstrated in a randomized controlled trial with 49 overweight and obese adolescents aged 9 to 15, that the mindfulness intervention group had an average BMI reduction of 1.18 kg/m^2^ after an 8-week intervention (p < 0.001) compared to the control group. In a study by Bernstein et al. (26), the mindfulness intervention group demonstrated a decrease in BMI percentile at a 1.5-year follow-up, while the control group did not experience significant changes. However, studies by Kumar et al. (27) and Shomaker et al. (28) found that there was no significant difference in BMI change between the mindfulness intervention group and the health education control group at the end of the intervention period. Researchers speculated that this could be due to the short intervention time and small sample size.

Additionally, WHR is an important indicator for measuring central obesity and is commonly used in the evaluation of adolescent obesity. Emmanouil et al. (29) conducted a randomized controlled trial involving 36 obese adolescents aged 9 to 13. The results demonstrated that the mindfulness intervention group experienced a significant reduction in WHR (p=0.008) compared to the control group, while there was no significant difference in BMI reduction. This suggested that although mindfulness intervention did not result in significant weight loss in adolescents, the decrease in WHR indicated that the distribution of fat shifted towards a healthier direction, which might be more crucial than weight loss in the short term. Research has indicated that abdominal fat deposition in children and adolescents is associated with an increased risk of cardiovascular metabolic diseases, highlighting the significance of monitoring this aspect (21). Nevertheless, with the exception of a few studies, the majority of existing research predominantly concentrated on changes in BMI among adolescents, with limited exploration of WHR. This could potentially serve as a promising area for future research.

### 3.2 The role of mindfulness in improving physiological indicators related to obesity

Existing research has discovered that mindfulness intervention can notably decrease physiological indicators related to obesity, including blood pressure, heart rate, serum cortisol, C-reactive protein, triglycerides, tumor necrosis factor, serum ghrelin, fasting blood sugar, and insulin levels. Pascoe et al. (22) conducted a Meta-analysis of 45 randomized controlled trials, revealing that mindfulness intervention not only lowered blood pressure and heart rate in participants but also reduced cortisol, C-reactive protein, triglycerides, and tumor necrosis factor, indicating the positive impact of mindfulness intervention on physiological indicators. However, the study analyzed a population that encompassed both adults and adolescents, and not all participants were overweight or obese. López-Alarcón et al. (30) conducted a randomized controlled trial with 45 obese adolescents aged 10 to 14, finding that in contrast to the control group that received conventional nutritional guidance, the mindfulness group experienced a significant decrease in serum ghrelin (p = 0.026) and serum cortisol levels (p=0.015) after an 8-week intervention, along with a significant reduction in BMI. This suggested that mindfulness can indirectly affect BMI through appetite or eating behavior. Ghrelin is a hormone that stimulates appetite, typically secreted to regulate food intake when energy supply is insufficient, but it may also be related to promoting the consumption of pleasurable foods (31). Lowering its levels can help control unhealthy eating behaviors. Cortisol is a stress-related hormone, and mindfulness intervention can reduce cortisol levels by reducing stress and weakening the activity of the hypothalamic-pituitary-adrenal axis, thereby reducing the effects of cortisol in metabolic tissues (liver, muscle, fat tissue) and ultimately resulting in weight loss (32). Shomaker et al. (33) discovered through a randomized controlled trial that mindfulness intervention can lower fasting blood sugar and fasting insulin levels (p=0.02) in overweight/obese girls aged 12 to 17 who were at risk of developing type 2 diabetes. This suggested that mindfulness intervention can prevent the onset of type 2 diabetes in overweight and obese adolescents by reducing insulin resistance.

### 3.3 The role of mindfulness in improving dietary choices and exercise habits and reducing impulsivity

Research has demonstrated that mindfulness intervention has a significant impact on enhancing dietary choices and exercise habits. Barnes et al. (19) conducted a randomized controlled trial with 40 adolescents aged 15 to 17 and found that the mindfulness intervention group exhibited significant improvements in dietary habits during a 6-month follow-up period after the intervention. The intake of low-calorie foods, foods without saturated fat, and foods low in saturated fat increased notably (by 7.7 servings, 5.1 servings, and 4.6 servings, respectively). The frequency of aerobic exercise also increased (>30 minutes of moderate-intensity aerobic exercise by 0.8 days/week, >20 minutes of high-intensity aerobic exercise by 1.4 days/week). Salmoirago-Blotcher et al. (34) discovered in a randomized controlled trial involving 53 ninth-grade students that the mindfulness intervention group showed increased physical activity compared to the control group that received conventional health education. However, this alteration was primarily noted in male students and those who had a prior exercise routine. There were no significant differences in dietary behaviors between the two groups (35). Nonetheless, there is a paucity of studies in this area, and additional research is needed to validate these findings.

Previous studies have demonstrated that adolescents with elevated levels of impulsivity and impaired emotion regulation are at a heightened risk of emotional eating and binge eating (24). As adolescents progress into adulthood, emotional eating can increase the likelihood of obesity and metabolic disruptions (36). Impulsivity and emotion regulation are crucial determinants of eating disorders, and these can be modified through behavioral interventions (37). Cotter et al. (23) conducted an open-label trial with 11 obese adolescents ranging in age 12 to 17, and it was discovered that after a 6-week mindfulness intervention, the participants experienced a significant reduction in impulsivity levels (assessed by their reaction time when selecting Go/No-Go responses to various images prompted by a computer program). Shomaker et al. (28) conducted a randomized controlled trial involving 54 obese adolescents aged 12 to 17. The results showed that the mindfulness intervention group had lower food reward sensitivity and less stress-induced eating behaviors during a 6-month follow-up after the start of treatment. Food reward sensitivity was determined by participants’ choice between a tasty snack and cash reward after completing a hypothetical task, while stress-induced eating was quantified by energy intake in a laboratory meal following stress induction. These findings suggested that mindfulness can help individuals remain composed when confronted with negative emotions, thereby reducing impulsivity, enhancing emotion regulation abilities, and ultimately reducing the likelihood of obesity.

### 3.4 The role of mindfulness in improving mental health outcomes

Mindfulness can also enhance the mental health of overweight or obese adolescents, which may increase their acceptance of dietary intervention and thereby lead to more favorable outcomes in obesity management. Stavrou et al. (25) conducted a randomized controlled study and discovered that overweight/obese adolescents in the mindfulness group experienced significant reductions in depressive and anxiety symptoms after an 8-week intervention compared to the control group. This was indicated by a decrease in scores on the Children’s Depression Inventory (p=0.004) and the Screen for Child Anxiety Related Emotional Disorders(p=0.04). Similarly, López-Alarcón et al. (30) determined that adolescents in the mindfulness group experienced decreased anxiety symptoms and perceived stress after an 8-week intervention compared to the control group. This was demonstrated by a reduction in scores (p<0.001) on the Spencer Anxiety Scale, which assesses social phobia, panic disorder, agoraphobia, generalized anxiety disorder, obsessive-compulsive disorder, separation anxiety disorder, and fear of bodily harm. In this study, both groups received calorie-restricted dietary intervention, but only the control group exhibited an increase in anxiety scores, which may be related to the potential negative impact of stress during the weight loss process. Therefore, this study suggested that applying mindfulness programs as a means to alleviate adolescent anxiety in obesity intervention could help prevent the occurrence of weight loss failures caused by increased anxiety under the pressure of dietary restrictions.

## 4 Choosing mindfulness intervention methods

The significant effects of mindfulness in adolescent obesity intervention provide a foundation for its implementation. Currently, the ways to intervene in adolescents’ mindfulness mainly encompass outpatient mindfulness therapy, delivering mindfulness courses in schools, short-video courses based on mobile internet, and family mindfulness therapy (23, 34, 38). Each of these approaches has its own advantages and disadvantages, serving as references for learning and refinement.

### 4.1 Outpatient mindfulness therapy

Incorporating mindfulness therapy rooms within pediatric obesity intervention clinics in hospitals is one of the most direct and viable approaches to implement mindfulness-based interventions. Cotter et al. (23) recruited 11 obese adolescents, ranging in age from 12 to 17, from patients at a pediatric weight management clinic in the United States. Through individual meetings with clinic physicians, these adolescents participated in a 6-week mindfulness intervention course, each session lasting 60 minutes. The course encompassed evidence-based breathing techniques and mindfulness-based dietary awareness training materials, emphasizing mindful eating, improving emotional responses, and cultivating non-judgmental awareness. Participants were required to complete approximately 10 minutes of brief homework exercises per day. All participants gave the mindfulness intervention course a full score (average 5 points, 100%), and the overall attendance rate for the 6 sessions was 85%. Their feedback included “Mindfulness helps me control my food intake and portion sizes”, “Helps me differentiate between true hunger and stress eating”, “I would recommend mindfulness to others after learning it”, “Helps me manage weight and emotions”, showing the feasibility and acceptability of outpatient mindfulness therapy. However, only 3 of the 11 individuals, attended the in-person clinic sessions, while the remaining 8 chose remote intervention, highlighting the significant advantages of remote medical care in terms of flexibility in time and location, which is worth of learning from.

### 4.2 Implementing mindfulness courses at school

Most existing studies implemented mindfulness interventions in schools, establishing mindfulness-related courses directly within schools to observe the preventive and control effects of mindfulness on adolescent obesity. These studies typically targeted the entire grade of students in the school for intervention, rather than solely the group of obese adolescents. Barns et al. (19) and Salmoirago-Blotcher et al. (34) conducted studies in schools, randomly assigning some ninth-grade adolescents from Georgia and Massachusetts in the United States to mindfulness intervention and control groups. School health education teachers taught mindfulness courses to the students. The former study demonstrated significant enhancements in diet and exercise habits in the mindfulness intervention group after the intervention, while the latter study observed an increase in physical activities in the intervention group, both with high attendance rates and acceptability. Krebs et al. (39) study recruited adolescents aged 9-12 from a primary school in New York City. After school every weekday, this school provided extracurricular activities such as arts and sports classes, and students could choose to participate voluntarily. Thus, researchers integrated mindfulness courses during this time period, allowing students to select them as an extracurricular activity. Eventually, students who chose the course became the intervention group, while those who did not participate in any extracurricular activities served as the control group, with a high course attendance rate. In summary, establishing mindfulness courses in schools can help reduce the additional burden on adolescents beyond academics, offering better convenience and feasibility. However, it may have limited specificity for the obese population and can thus be used as part of health education for the prevention of adolescent obesity.

### 4.3 Short video courses based on mobile internet

Although screen time has been recognized as a risk factor for adolescent obesity (40), educational interventions for obese patients using online seminars and videos have demonstrated positive outcomes (41). Moreover, a study (42) discovered that diet counseling via videos was effective for weight loss during the COVID-19 pandemic. In addition, video sports games have been shown to assist in reducing the weight of overweight children (43). Due to the busy academic schedule of adolescents and difficulties in conducting offline courses, some studies have investigated the utilization of short-video courses based on mobile internet to enhance the feasibility and acceptability of mindfulness intervention. Turner et al. (38) developed and evaluated an independent mindfulness-based app, requiring 15 adolescents aged 14-18 to watch a guided mindfulness practice short video through the app daily for 6 weeks. The app received high ratings and daily usage rates after the intervention. Given the academic pressure on Chinese adolescents and the extensive use of smartphones, it can be assumed that this intervention method is more suitable for implementation in China. Zhang et al. (44) recruited 55 obese adolescents (BMI≥24kg/m^2^) aged 16-18 from three high schools in Beijing and Nanjing in China. Over a period of 3-week period, they received 10 short videos via WeChat on mindfulness and its practices, each ranging 3-10 minutes in duration, with unlimited access. After the intervention, participants submitted assessment reports in the fourth week. The participants experienced significant weight loss after the intervention, with a decrease in the frequency of unhealthy food consumption, emotional eating, external eating cues, and cravings for specific foods. Their mindfulness awareness in eating and self-efficacy in diet significantly improved. 84% of adolescents who adhered to the full mindfulness intervention program achieved significant results, indicating that providing short video mindfulness intervention for Chinese adolescents is feasible and effective.

### 4.4 Family mindfulness therapy

Adolescents are highly influenced by family dietary habits and lifestyles, prompting research on family-based mindfulness therapy that involves parents in the intervention. Kumar et al. (27) randomly assigned 22 obese adolescents aged 14-17 and their parents to a mindfulness intervention group and a standard dietary control group. The intervention group participated in four 90-minute mindfulness sessions over 10 weeks, with adolescents and their parents attending separate classes in different rooms simultaneously. Compared to the control group, adolescents in the intervention group demonstrated enhancements in diet-related mindfulness awareness (assessed using MEQ scores) and dietary self-efficacy (measured using WEL scores) at a 2-year follow-up (p=0.01). The attendance rate for all mindfulness intervention sessions was 100%, indicating that family-based mindfulness therapy for obese adolescents is feasible and highly accepted.

## 5 Limitations and future prospects

This article summarizes the impact of mindfulness on improving both physiological and psychological indicators of adolescent obesity, while also presenting various options for implementing mindfulness therapy. However, it acknowledges certain limitations. Firstly, most existing studies are single-center, small-sample investigations that employ different evaluation methods and indicators, leading to inconsistent conclusions. Therefore, there is a need for multi-center, large-sample studies to clarify the findings of each research effort. Secondly, much of the existing research has not examined the mechanisms through which mindfulness interventions impact adolescent obesity, indicating a need for further studies to clarify these specific mechanisms. Additionally, only a limited number of studies have been conducted in the Asian region, raising concerns about the applicability of these conclusions to Chinese and other Asian populations.

Cultural differences contribute to varying dietary habits, and exercise routines among people from different countries. In terms of dietary habits, Chinese individuals typically emphasize rice and a variety of vegetables, while consuming fewer high-calorie and high-fat foods (45). Regarding exercise, many Chinese people engage in leisure-time physical activity (46). Consequently, there is an urgent need for relevant research in China to explore mindfulness intervention methods tailored to the cultural background and characteristics of Chinese adolescents, ultimately providing more effective treatment strategies for obese children and those at risk of obesity.

## 6 Conclusions

Mindfulness, as an emerging therapeutic approach, has been recognized to play a unique role in adolescent obesity intervention in recent years. This article reviewed the research progress of mindfulness in adolescent obesity intervention, summarized the effects of mindfulness on reducing BMI and WHR, improving obesity-related physiological indicators, enhancing dietary choices and exercise habits, reducing impulsivity and improving eating disorders, as well as improving psychological health outcomes, and analyzed some of its mechanisms of action. In terms of the selection of mindfulness therapy forms, outpatient mindfulness therapy, school mindfulness courses, mobile internet short video courses, and family mindfulness therapy were all worthy of our reference and learning. These studies supported the preliminary effectiveness of mindfulness in adolescent obesity intervention. In the future, cross-cultural research could be conducted to clarify how cultural differences influence the effectiveness of mindfulness interventions for adolescent obesity. Additionally, investigating the differential effects of various mindfulness approaches—such as mindfulness for healthier eating versus mindful eating—may emerge as a promising avenue for research.
